# The Potential for Early Health Economic Modelling in Health Technology Assessment and Reimbursement Decision-Making Comment on "Problems and Promises of Health Technologies: The Role of Early Health Economic Modeling"

**DOI:** 10.15171/ijhpm.2020.17

**Published:** 2020-02-05

**Authors:** Hansoo Kim, Stephen Goodall, Danny Liew

**Affiliations:** ^1^School of Public Health and Preventive Medicine, Monash University, Melbourne, VIC, Australia.; ^2^Centre for Health Economics Research and Evaluation, University of Technology Sydney, Ultimo, NSW, Australia.

**Keywords:** Early Assessment, Health Economic Modelling, Reimbursement, Health Technology Assessment, Australia

## Abstract

Grutters et al recently investigated the role of early health economic modelling of health technologies by undertaking a secondary analysis of health economic modelling assessments performed by their group. Our commentary offers a broad perspective on the potential utility of early health economic modelling to inform health technology assessment (HTA) and decision-making around reimbursement of new health technologies. Further we provide several examples to compliment Grutters and colleagues’ observations.

## Introduction

Health technology assessment (HTA) is routinely used to support reimbursement decisions. The process typically involves health economic modelling of available efficacy and resource utilisation data on health technologies. However, the role is less clear for *early* health economic modelling to identify potentially cost-effective new technologies during their development phases; that is, prior to robust efficacy data being available. Grutters et al recently investigated this issue by undertaking a series of case studies of 32 health economic modelling assessments of 30 innovations performed by their group.^1^ There were some limitations to the study, mainly stemming from the authors drawing a small sample of unpublished HTAs (n = 32) from a single group (their own), and considering only the perspective of the Dutch healthcare system. Nonetheless, their conclusion that early assessment provided insight into the potential cost-effectiveness and uncertainty associated with the technology highlights an important point: that any intelligence on the future market for a new health technology is valuable, not only for its sponsors, but also payers as well as providers and patients even though the these have different informational needs.

Early consideration of the cost-effectiveness of a new health technology is a logical step, given its prominence in reimbursement decisions. The present commentary offers a broad perspective on the potential utility of early health economic modelling to inform HTA and decision-making around reimbursement of new health technologies.

First, it is important to recognise that there is significant variation in HTA requirements from country to country,^2,3^ and not all HTA agencies even require health economic modelling to inform decision-making. As such, it can be expected that the usefulness of early health economic modelling will depend on the market within which it is undertaken, as well as the rules for HTA in that market.

In HTA jurisdictions like Australia, Canada and the United Kingdom, there is formal requirement for health economic modelling, and although there are well established guidelines for undertaking this, there is generally also acceptance of novel approaches to accommodate innovative therapies. For example, the advent of immuno-oncology agents, which have unique biological mechanisms of action and clinical effects, has necessitated new approaches to economic evaluation, especially with regards to extrapolating and translating data beyond the pivotal clinical trials.^4^

In countries without mandatory health economic modelling, like South Korea and Taiwan, the utility of early modelling is less clear. However, this strategy can still be used to characterise both the clinical and economic environments within which the new health technology will be assessed for reimbursement. Insights could be gained into the unmet clinical need, the extent to which the health technology would address this, and the price at which this could be done in a cost-effective manner. Hence although formal early health economic modelling may not be mandated for stop/go decisions with respect to reimbursement, the exercise would still be very informative.

Regardless of the jurisdiction, there are three key areas in which early health economic modelling offers the most potential in HTA and reimbursement decision-making: identifying uncertainty, assist in the generation of real world evidence and informing risk-share agreements.

## Minimising Uncertainty

Parameter uncertainty and structural uncertainty in economic evaluations is a major problem, and a common reason for failure of reimbursement applications.^5^ Early health economic modelling provides a means to identify input that will contribute most to the parameter uncertainty of a cost-effectiveness analysis. For example, early modelling with deterministic or probabilistic sensitivity analyses may demonstrate that the relative efficacy (such as the hazard ratio associated with an outcome) of the new health technology will exert the greatest influence on the likelihood of its cost-effectiveness, even after taking into account other key input parameters. Alternatively, it could identify thresholds in the risk of the target disease at which an intervention is most likely to be effective, and hence cost-effective and aid in calibration of the model structure. This type of information could inform the ongoing clinical development of the new health technology, and/or recommend further research to minimise parameter uncertainty.

Another common source of uncertainty lies in the long-term benefits of health technologies. In most HTAs, it is necessary to extrapolate outcome data beyond the duration of clinical trials, which are typically short (and may be surrogate). The Australian Pharmaceutical Benefits Advisory Committee (PBAC)^6^ and the National Institute for Health and Care Excellence^7^ recommend using visual inspection of several possible distributions such as the Weibull, loglogistic, lognormal, Gompertz and exponential as well as comparisons of the Akaike information criteria (AIC) when choosing distributions for extrapolations. The guidelines stipulate that the distribution with lowest AIC score should be chosen.

The AIC is agnostic to the underlying behavior in the data and the mathematical properties of the distributions. Sometimes the AIC values can be almost identical as reported by Bohensky et al^8^ where the AIC for the Weibull distribution was observed to be 243.204 and 243.451 for the loglogistic distribution in the post progression health state. This equates to a 0.1% difference but more importantly the two distributions are fundamentally different. The Weibull distribution is monotone and the hazard cannot change direction. This means that if an increasing hazard (eg, risk of death) is assumed then it will keep increasing over time. The loglogistic distribution on the other hand allows for the hazard to change direction over time.^9,10^ For some of the immune-oncology drugs it is well-known^11^ that after an initial steep drop in the survival curve there is a plateau like effect meaning that the hazard increases to begin with but then decreases after a while.

Early modelling would be able to assist in validating the behavior of the hazard and guide modelers in the choice of distribution instead of relying on measures like the AIC.

Another issue is time horizon of the extrapolation. For example, in Australia, trastuzumab for patients with HER2 positive early breast cancer following surgery in association with chemotherapy was considered for reimbursement by the PBAC in July 2006. The sponsor had extrapolated outcome data from the clinical trial over a 40-year time horizon, but not detailed the methods for extrapolation.^12^ This was a major source of uncertainty. There were no explicit guidelines regarding the extrapolation of observed data but this was a major source of uncertainty. However, since then, the topic has been widely debated^13,14^ and current guidelines now mandate rigorous examination of extrapolation methods.^6^ Had early health economic modelling been undertaken as part of the HTA for trastuzumab, the multiple sources of uncertainty would have been identified, including timeframes over which efficacy measures could have been assumed, the various functions that could have been fit to extrapolate survival data, and the impact of decreasing adherence over time.

Underlying uncertainty relating to the structure of the model (structural uncertainty) such as how health states are linked in a Markov model or choice of underlying survival functions in a partitioned survival model can also be minimised through early health economic modelling. This can be done by ensuring that economic models are set up in flexible ways to allow for testing of different scenarios. These scenarios could relate to key structural assumptions that are not normally taken tested in models such as Markovian assumptions surrounding time dependent transition probabilities, or what time point to apply extrapolation to Kaplan Meier data in partitioned survival models. Key to this is the mapping of future treatment pathways for the particular diseases of interest. Predicting future treatment pathways is challenging, but achievable with available data and expertise. For example, a review of clinical trial registries like www.clinicaltrials.gov and the convening of expert advisory panels are both useful. It is important to note that future comparators may not be the same modality; for example, what is a drug comparator now may be a device in the future.

## Generation of Real-World Data

The use of real-world data is an area of major increasing interest in HTA.^15^ Early health economic modelling uses real-world data in conjunction with clinical trial data, and is potentially a useful tool that can used to aid and guide gathering of real world data. An example of real-world data and early modelling guiding decision-making can be found in the work by Tappenden et al in 2017.^16^ Here, registry data, evidence drawn from the literature and expert opinion were used to populate an early model on an adherence intervention to improve outcomes for patients with cystic fibrosis. The analysis allowed for estimation of health gains and expected costs savings over a five-year period and the study is still ongoing.

Clinical trials are typically designed with the aim of obtaining regulatory approval for the health technology, not reimbursement approval. Moreover, due to patient selection and strict protocols, clinical trials can often overestimate an interventions effect when implemented into clinical practice. Thus, a major issue in HTA is whether efficacy data from a clinical trial translates into real-world effectiveness.^17^ Early modelling is a vehicle for translating and synthesizing efficacy data from early phase clinical trials into real-world effectiveness data and can therefore support future reimbursement decisions. A major advantage of modelling is that multiple and complex scenarios can be explored with currently available modelling techniques. For example, agent-based systems can take into consideration that many clinicians do not prescribe drugs exactly as per reimbursement criteria, and patients are often not compliant with the intended regimen.^18^ Other examples include dynamic simulation of systems and discrete event simulations.^19^

Furthermore, early health economic modelling could aid the generation of real-world data when no other data are available. This would typically be the case when clinical trials have not yet been reported. Real-world data such as clinical registries, patient charts and script data can easily be synthesised using a health economic model. This would then enable additional research recommendations to be based on many different types of evidence particularly health resource utilisation data, which is often protocol driven in clinical trials.

## Risk Share Agreements

There is currently increased focus on how to accelerate access to new health technologies. Initiatives include streamlining processes between regulatory and HTA authorities,^20^ as well as through harmonisation initiatives like the European Network for Health Technology Assessment.^21^ Moreover, there seems to be a tendency for payers to be to prepared to enter into ‘coverage with evidence’ development schemes or risk share agreements.^4,22^ This is typically done in order to acquire further data to support the evidence for the incremental cost-effectiveness ratio. Unfortunately, while these schemes and agreements offer a solution to early funding as reimbursement is granted even though evidence such as mature survival data is not yet available, the risks are often not well-understood.^23,24^ Early health economic modelling could bridge this gap by better conceptualising the risks and uncertainties for both payers and sponsors.

Early modelling would have been valuable in the high unmet need case of ipilimumab for advanced melanoma in Australia. After three failed initial applications by the sponsor for reimbursement, the Australian PBAC finally granted coverage of ipilimumab under a condition that the sponsor to provide future evidence of improved overall survival.^25^ A post marketing follow-up program was established and the overall survival claim was verified.^26^ Early health economic modelling with input from the payer could have identified the overall survival claim as a major issue up front, thereby avoiding multiple submissions.

Many risk share agreements are still financial in nature,^27^ which is another area where early modelling can be valuable. For example, the Australian PBAC determined that a financial risk share agreement for pembrolizumab in first line treatment of non-small cell lung cancer was needed.^28^ A 100% rebate beyond a subsidisation cap was proposed to mitigate the overall budgetary risk to the government if the number of actual patients exceeded that which was agreed upon. Early economic modelling could have been used to inform the budget modelling and subsequently offer alternatives to this arrangement, in which all the risk is carried by one party.

## Conclusion

Early health economic modelling provides a mechanism for early assessment of new health technologies, as pointed out by Grutters et al. Its acceptance and utility will depend on the environment and context within which it is undertaken, but minimising uncertainty, generation of real-world evidence and informing risk share agreements stand out as areas of greatest potential. There is unfortunately a lack of literature demonstrating the power of early modelling and researchers from academia and industry are encouraged to publish more papers describing health economic modelling before/during/after development of health technologies.

It would be surprising if pharmaceutical and device industry did not already use early modelling in some form to inform development of their products.^29^ However, through wider collaboration with stakeholders and payers, early health economic modelling could be instrumental in bridging the gap from laboratory to patient access in a more seamless way.

## Ethical issues

Not applicable.

## Competing interests

Authors declare that they have no competing interests.

## Authors’ contributions

All authors contributed to the conception, drafting and approval of manuscript.

## Authors’ affiliations

^1^School of Public Health and Preventive Medicine, Monash University, Melbourne, VIC, Australia. ^2^Centre for Health Economics Research and Evaluation, University of Technology Sydney, Ultimo, NSW, Australia.
